# Adding acute treatments for patients on triptans: Results of the American Migraine Prevalence & Prevention (AMPP) study

**DOI:** 10.1186/1129-2377-1-S14-P214

**Published:** 2013-02-21

**Authors:** RB Lipton, D Serrano, S Kori, C Cunanan, AN Manack, ML Reed, DC Buse

**Affiliations:** 1 Albert Einstein College of Medicine, USA; 2 Vedanta Research, USA; 3 MAP Pharmaceuticals, USA; 4 Allergan Inc., USA

## Background

Augmenting a triptan regimen with another acute medication may be an indicator of suboptimal treatment.

## Objectives

To quantify changes in headache-related disability for migraineurs who added an acute treatment to an existing triptan regimen in a population-based sample.

## Methods

AMPP study surveys were mailed to a sample of 24,000 persons with "severe headache" identified in 2004 and followed annually through 2009. Eligible subjects had ICHD-2 migraine treated with a triptan one year and data in the subsequent year (a couplet). We examined 4 patterns of treatment: (a) consistent triptan treatment, (b) adding another triptan, (c) adding an opioid or barbiturate or (d) adding an NSAID. Change in disability was measured by MIDAS from the second to the first year (negative change scores reflect improvement). Change scores were modeled via ANOVA for all couplets and for 3 average headache-day frequency strata: low (0-4 days/month), moderate (5-9 days/month), and high frequency episodic/chronic migraine ([HFEM/CM]≥10 days/month). ANOVAs were estimated for each possible adding pattern relative to the consistent triptan use group. The values of (b) represent change in MIDAS score.

## Results

327 respondents met inclusion criteria and reported an add pattern of interest. Adding another triptan was significantly associated with increased headache-related disability (b=10.4, p=.01) over one year, as was adding an NSAID. The NSAID effect was greatest in those with HFEM/CM compared to those with 0-4 days/month (b=24.1, p=.03), and even greater for those with HFEM/CM compared to 5-9 days/month (b=29.3, p=.02). Adding an opioid or barbiturate was not significantly associated with changes in disability.

## Conclusion

Adding an opioid or barbiturate was not associated with significant change in headache-related disability; however, adding a triptan or an NSAID was associated with increased disability. The effects for NSAIDs were greatest among the HFEM/CM group. Improvement in options for migraine management is needed, especially for persons with high frequency headache.

